# Genetic diversity of trypanosome species in tsetse flies (*Glossina* spp.) in Nigeria

**DOI:** 10.1186/s13071-019-3718-y

**Published:** 2019-10-14

**Authors:** Judith Sophie Weber, Sen Claudine Henriette Ngomtcho, Stephen Saikiu Shaida, Gloria Dada Chechet, Thaddeus Terlumun Gbem, Jonathan Andrew Nok, Mohammed Mamman, Daniel Mbunkah Achukwi, Sørge Kelm

**Affiliations:** 1 Centre for Biomolecular Interactions, Department of Biology and Chemistry, University of Bremen, Bremen, Germany; 2 Department of Biological Sciences, University of Ngaoundéré, Ngaoundéré, Cameroon; 3 Ministry of Public Health, Yaoundé, Cameroon; 4 Nigerian Institute for Trypanosomiasis Research, Kaduna, Nigeria; 5 Department of Biochemistry, Ahmadu Bello University, Zaria, Nigeria; 6 Africa Centre of Excellence for Neglected Tropical Diseases and Forensic Biotechnology, Ahmadu Bello University, Zaria, Nigeria; 7 Department of Biology, Ahmadu Bello University, Zaria, Nigeria; 8 TOZARD Research Laboratory, Bambili-Tubah, Cameroon

**Keywords:** Trypanosomes, *T. grayi*, Genetic diversity, Trypanosomiasis, Nigeria

## Abstract

**Background:**

Trypanosomes cause disease in humans and livestock in sub-Saharan Africa and rely on tsetse flies as their main insect vector. Nigeria is the most populous country in Africa; however, only limited information about the occurrence and diversity of trypanosomes circulating in the country is available.

**Methods:**

Tsetse flies were collected from five different locations in or adjacent to protected areas, i.e. national parks and game reserves, in Nigeria. Proboscis and gut samples were analysed for trypanosome DNA by molecular amplification of the internal transcribed spacer 1 (ITS1) region and part of the trypanosome specific glycosomal glyceraldehyde-3-phosphate dehydrogenase (*gGAPDH*) gene.

**Results:**

The most abundant *Trypanosoma* species found in the tsetse gut was *T. grayi*, a trypanosome infecting crocodiles. It was ubiquitously distributed throughout the country, accounting for over 90% of all cases involving trypanosomes. *Trypanosoma congolense* was detected in gut samples from all locations except Cross River National Park, but not in the proboscis, while *T. brucei* (*sensu lato*) was not detected at all. In proboscis samples, *T. vivax* was the most prominent. The sequence diversity of *gGAPDH* suggests that *T. vivax* and *T. grayi* represent genetically diverse species clusters. This implies that they are highly dynamic populations.

**Conclusions:**

The prevalence of animal pathogenic trypanosomes throughout Nigeria emphasises the role of protected areas as reservoirs for livestock trypanosomes. The genetic diversity observed within *T. vivax* and *T. grayi* populations might be an indication for changing pathogenicity or host range and the origin and consequences of this diversity has to be further investigated.
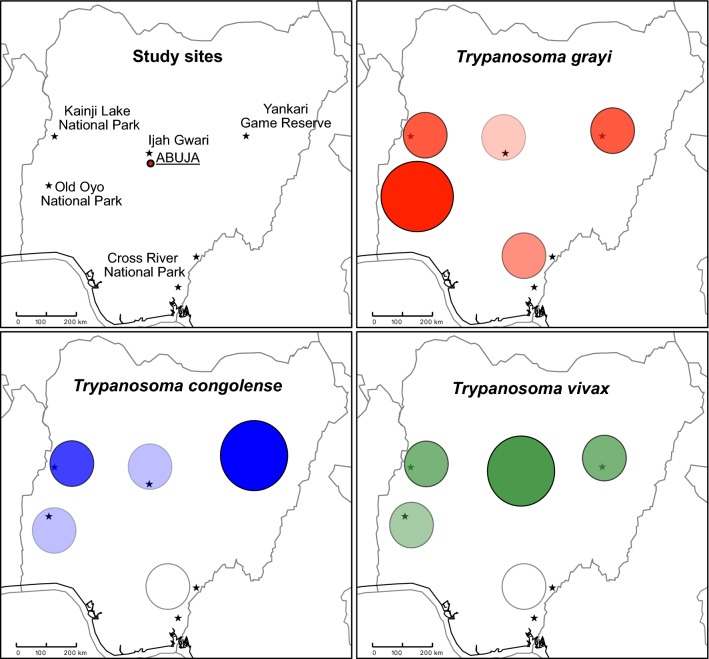

## Background

African trypanosomes are protozoan parasites causing disease in humans and livestock in sub-Saharan Africa. The trypanosomes of greatest socioeconomic importance are *Trypanosoma brucei* (*sensu lato*), *T. congolense* and *T. vivax*. They rely largely on the presence of tsetse flies (*Glossina* sp.) as their key biological vector, whose geographical distribution is restricted to sub-Saharan Africa. *Trypanosoma vivax* can also be mechanically transmitted by other biting flies such as tabanids and *Stomoxys* [1–3].

Trypanosomes are separated into salivarian and stercorarian parasites. The Salivaria are transmitted *via* the saliva and mouthparts of the infected vector and consist of the African trypanosomes *Trypanosoma brucei* (*s.l.*), *T. congolense*, *T. simiae*, *T. godfreyi*, *T. suis* and *T. vivax*. *Trypanosoma brucei* (*s.l.*) is of special medical importance, as the subspecies *T. b. gambiense* and *T. b. rhodesiense* cause the severe disease human African trypanosomiasis (HAT; sleeping sickness). *Trypanosoma congolense*, *T. vivax* and the third subspecies of *T. brucei* (*s.l.*), *T. b. brucei*, cause African animal trypanosomiasis (AAT; nagana) mainly in cattle and other livestock [4]. *Trypanosoma congolense* and *T. brucei* (*s.l.*) first colonise the midgut before migrating to the proboscis and salivary glands of tsetse, respectively, whereas *T. vivax* directly migrates from the crop *via* the proventriculus to the proboscis [5, 6]. They are transmitted to the mammalian host during blood-feeding.

Stercorarian trypanosomes are transmitted *via* the faeces of blood-sucking insects, where they develop in the mid- and hindgut. Examples of this group are *T. grayi*, an African trypanosome infecting crocodiles [7, 8], and *T. theileri*, a worldwide distributed trypanosome infecting cattle and other ungulates [1, 9].

Trypanosomiasis is highly prevalent in sub-Saharan Africa. Nigeria is the largest economy and most populous country in Africa with an estimated population of over 190 million people in 2016 [10]. In many regions in Nigeria, people depend largely on farming and livestock rearing. Especially in remote rural regions, humans and livestock are at risk of coming into contact with infected tsetse. Reported HAT cases were low in Nigeria during the last decade, with a maximum of three newly recorded cases per year [11]. However, cases might remain unreported in rural regions of the country with restricted health surveillance. On the other hand, AAT is known to be highly prevalent. A meta-analysis of reports on AAT in Nigeria obtained over the last six decades (1960–2017) revealed a continuous burden of the disease in cattle and prevalence of the parasites in tsetse [12]. Recent studies that relied on molecular identification of trypanosomes in tsetse [13–15] and cattle [16, 17] found *T. vivax* and *T. congolense* to be the most prevalent trypanosomes in different areas of Nigeria. Prevalence of *T. b. brucei* was generally low compared to *T. vivax* and *T. congolense*. The only exception is provided by a study in southern Nigeria where it was highly prevalent in tsetse (> 50%) [14], while in northern Nigeria this parasite was not detected at all [13]. To our knowledge, an overview of parasites present in different tsetse populations from different regions in Nigeria collected within the same time and analysed with the same molecular methods has not yet been undertaken. Therefore, we collected tsetse from five sites located throughout Nigeria. The focus was largely on national parks (NP) and game reserves (GR) to investigate the role of protected areas as reservoirs for trypanosomes. Tsetse fly gut and proboscis samples were molecularly analysed to investigate the trypanosome species diversity and their genetic diversity within these locations.

## Methods

### Tsetse trapping

**Fig. 1:**
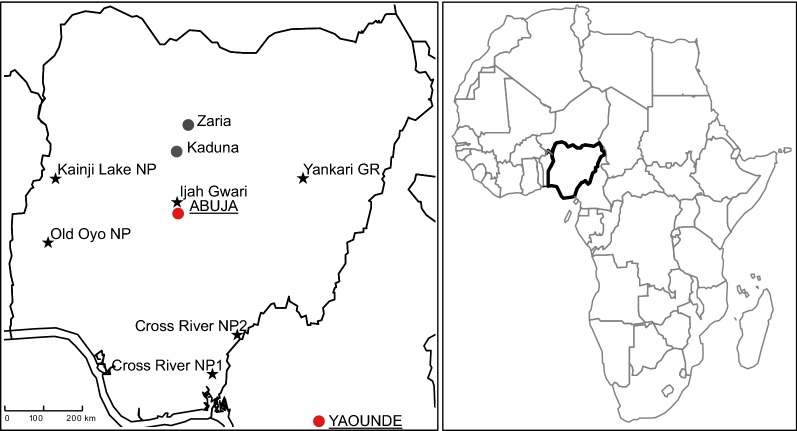
Location of tsetse trapping sites in Nigeria. Trapping sites are marked with stars. Description of the collection sites can be found in [18]. *Abbreviations*: Cross River NP 1, Cross River National Park Akampka division; Cross River NP 2, Cross River National Park Butateng division; GR, Game Reserve; NP, National Park

Tsetse fly trapping was carried out in March 2014 during the end of the dry season. Ijah Gwari (Niger State) was revisited for a second collection in November 2014 during the beginning of the dry season. Results obtained from both surveys in Ijah Gwari were pooled. A description of the trapping sites can be found in Shaida et al. [18]. The locations of all sites are displayed in Fig. 1.

Trapping of adult tsetse was performed as described [18]. Briefly, standard biconical traps [19] (Vestergaard-Frandsen, Lausanne, Switzerland) were set up for at least 48 h at each sampling site before relocation. Flies were collected every 24 h and transported in cool boxes to the base camp for sorting, morphological species identification and dissection.

### Dissections and tissue preparation

Tsetse tissues were dissected as previously described [18]. Forceps and microscope slides were decontaminated in 70% ethanol and acetone between dissections. Briefly, the proboscis was removed and carefully immersed in 200 µl of DNA/RNA preservation agent NAPA (25 mM sodium citrate, 10 mM ethylenediaminetetraacetic acid (EDTA), 5.3 M ammonium sulfate, pH 7.5 [20]). Gut tissue was dissected out last and homogenised in 200 µl of 50 mM Tris-Cl, pH 9.0, using four 2.38 mm metal beads (MoBio Laboratories, Carlsbad, CA, USA). Fifty microlitres of the homogenate was added to 500 µl of NAPA. All samples were kept at 4 °C or frozen if possible until brought back to the laboratory.

### DNA purification

DNA was purified from homogenised gut tissue in NAPA with the DNeasy Blood and Tissue Kit (Qiagen, Hilden, Germany) according to the instructions of the manufacturer with the following adjustments: 100 µl of homogenate was used for purification and eluted with the same volume of elution buffer. DNA content was checked by spectrophotometric detection at 260 nm.

DNA from the proboscis was crudely extracted by a prolonged proteinase digest with 0.33 mg/ml Proteinase K (Thermo Fisher Scientific, Dreieich, Germany) in 10 mM phosphate buffer, pH 7.4 for 1 h at 55 °C, following heat inactivation of the enzyme for 45 min at 80 °C.

### Trypanosomal detection

A nested PCR targeting the ITS1 region of Kinetoplastida was used for the detection of trypanosomes present [20, 21]. The method allows detection down to 100 trypanosomes within one sample [21]. For gut samples, 5 µl of purified template DNA was used. This corresponds to 0.25% of original tsetse gut tissue and allows on average the detection of a parasite load higher than 4 × 10^4^ parasites present in the tsetse gut. In the case of the crude proboscis samples, 1 µl of template was used in the first reaction. For both tissues, the first reaction was diluted 1:80 and 1 µl of the dilution used in the second reaction. In both reactions, the mastermix consisted of 200 µM dNTPs, 1× DreamTaq Green Buffer, 2.5 U DreamTaq Polymerase (all Thermo Fisher Scientific) and 2 µM of each primer (Sigma-Aldrich, Darmstadt, Germany) in a final volume of 25 µl. The PCR cycle for both reactions was as follows: initial denaturation at 95 °C for 3 min; 30 cycles of 1 min at 94 °C, 30 s at 54 °C and 30 s at 72 °C; final elongation for 5 min at 72 °C.

The annealing temperature was set to 54 °C for *T. congolense* and *T. grayi* species-specific PCR. *Trypanosoma grayi*-specific primers require a final concentration of 4 mM MgCl_2_ in both reactions [20]. Amplicons were separated on 2% TBE (Tris-Borate-EDTA buffer) agarose gels at 100 V and stained with Stain-G (Serva, Heidelberg, Germany). Size estimation was based on a low molecular weight marker, GeneRuler 50 bp DNA ladder (Thermo Fisher Scientific).

A nested PCR targeting the *gGAPDH* of trypanosomes was used for additional confirmation of trypanosomal detection and phylogenetic analysis. Primers were adapted from Hamilton et al. [22] and one inner primer designed in this study (Table 1). The mastermix was the same as the one for ITS1 detection, except for the use of standard Dream*Taq* buffer (Thermo Fisher Scientific). Due to the lower sensitivity of the reaction, 7 µl of gut template and 5 µl of proboscis sample were added to the first reaction, and 5 µl of the first reaction was used undiluted in the second reaction. The first reaction was started with denaturation for 3 min at 95 °C, followed by 30 cycles of 1 min at 95 °C, 30 s at 55 °C and 1 min at 72 °C, and a final elongation at 72 °C for 10 min. The second reaction was performed with the same parameters, except the annealing temperature was lowered to 52 °C.

**Table 1:** Primer sequences used in this study and their sources

Primer name	Nucleotide sequence (5′–3′)	Amplicon size (bp)	Source
ITS1-out2	CTTTGCTGCGTTCTT	Variable (100–650)	[21]
ITS1-out1	TGCAATTATTGGTCGCGC		
ITS1-in1	TAGAGGAAGCAAAAG		
ITS1-in2	AAGCCAAGTCATCCATCG		
gGAPDH-out	TTYGCCGYATYGGYCGCATGG	816	[22]
gGAPDH-out	ACMAGRTCCACCACRCGGTG		
gGAPDH-in	GCSTAYCAGATGAAGTACGAC		This study
gGAPDH-in	GTTYTGCAGSGTCGCCTTGG		[22]
TGR-out1	TGGCAGACACATACCTGCCA	526	[20]
TGR-out2	TGGGGATTACGGATGAAAC		
TGR-in1	TTAAGGAGGCGCTCAGGTTC		
TGR-in2	TGTGCATATACGTCTATG		
TCON-out1	TGCAATTATTGGTCGCGC	Variable (681–781)	[20]
TCON-out2	TGTTGGTCGACACTGAGA		
TCON-in1	TCGCGTGTCTCACGT		
TCON-in1	TCAAAGATTGGGCAATGT		

Amplification products were separated in a 1.5% TAE (Tris-Acetate-EDTA buffer) agarose gel and stained as described above.

Differences in trypanosome distribution were tested for significance using the Chi-square test in Prism 8. A *P*-value < 0.05 was considered significant. The 95% confidence interval was calculated using Prism 8.

### Sequencing of PCR products

PCR amplification products were excised from the gel and purified using the GeneJET gel purification kit (Thermo Fisher Scientific). The purified DNA was then used for direct Sanger sequencing by a commercial provider (Microsynth SeqLab, Göttingen, Germany). In the case of *gGAPDH*, this was sufficient in all cases. For ITS1 fragments, subcloning was necessary in several cases, especially when the DNA amplicon was smaller than 400 bp. In these cases, purified DNA fragments were cloned into pJET1.2 using the ClonJET PCR cloning kit (Thermo Fisher Scientific). Plasmids were purified and then sent for Sanger sequencing as stated above.

### Sequence comparison and phylogenetic analysis

Obtained sequences were cleaned and trimmed using Geneious Pro v.5.5.9 [23]. They were aligned using the Geneious Alignment tool. *gGAPDH* sequences were aligned with Gap open penalty 15 and Gap extension penalty 5 to avoid the introduction of gaps in the protein coding sequence. *gGAPDH* sequences were translated using the translation tool from Geneious and the translation confirmed by BLAST analysis against the GenBank database.

Phylogenetic relationships were analysed using MEGA v.6 [24]. The alignments were imported from Geneious, and Neighbour-Joining trees of *gGAPDH* sequences calculated using complete deletion of gaps with 700 bootstrap replications for all nucleotides. The same parameters were used for the translated protein sequences.

## Results

A total of 424 live tsetse were collected from five different sites throughout Nigeria (Fig. 1, Additional file 1: Table S1). From 23 of these flies, only gut or only proboscis samples were available, respectively, and a total of 413 gut samples and 412 proboscis samples were obtained for molecular analysis of *Trypanosoma* prevalence.

### Distribution of *T. grayi* and other *Trypanosoma* species in Nigeria

Evidence for trypanosomal DNA was obtained by amplification of the ITS1 region of Kinetoplastida in 42% (95% CI: 37.4–46.9%) of the 413 analysed gut samples and 18.7% (95% CI: 15.2–22.7%) of the 412 proboscis samples (Tables 2, 3). Note that the prevalence detected here does not necessarily represent the number of fly infections, as molecular analysis does not differentiate between ongoing infections and residual DNA from cleared infections. *Trypanosoma* species were initially assigned according to ITS1 amplicon lengths [20, 21]. Species identification was further confirmed by sequencing of representative ITS1 amplicons or by additional sequencing of part of the *gGAPDH* gene [22] (for details on analysed samples and sequences, see Additional file 1: Table S1).

**Table 2:** Prevalence (in %) of *Trypanosoma* species in the different collection sites in the tsetse gut

	All	Yankari GR	Kainji Lake NP	Old Oyo NP	Cross River NP	Ijah Gwari
*T. grayi*	37.2 (32.7–42.0)	20.0 (10.9–33.8)	30.6 (22.7–39.8)	55.3 (48.2–62.2)	14.3 (5.0–34.6)	6.0 (1.6–16.2)
*T. congolense*	4.8 (3.2–7.4)	8.9 (0–17.5)	7.4 (0–12.5)	3.2 (0–5.8)		4.0 (0–10.4)
*T. vivax*	1.0 (0.4–2.5)		2.8 (0.8–7.9)		4.8 (0.2–22.7)	
*T. theileri*	0.5 (0.1–1.7)		1.9 (0–4.9)			

*Notes*: *Trypanosoma* species were assigned according to ITS1 size [20, 21] and confirmed by sequencing of selected ITS1 and gGAPDH amplicons [22]. The lower and upper limits of the 95% confidence interval are indicated in parentheses

The most prevalent *Trypanosoma* species encountered in this study were *T. grayi* and *T. congolense* in gut tissue and *T. vivax* and unexpectedly *T. grayi* in proboscis. A comprehensive overview of *Trypanosoma* species detected in each sampling site is shown in Additional file 2: Figure S1.

### Trypanosome distribution in tsetse gut samples

**Fig. 2:**
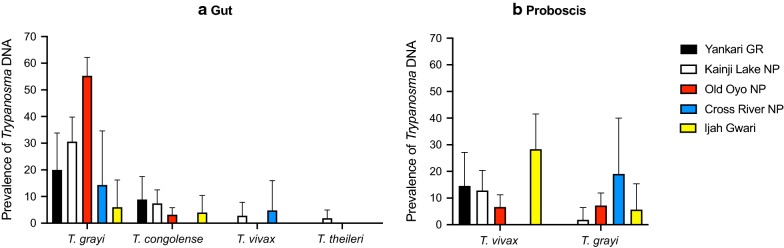
Relative prevalence of *Trypanosoma* DNA in tsetse gut (**a**) and proboscis (**b**) samples from four sampling sites in Nigeria. *Trypanosoma* species were assigned according to ITS1 size estimation [20, 21] and confirmed by sequencing of ITS1 and *gGAPDH* [22]. All cases of one *Trypanosoma* species were included in the species count irrespective of the presence of any other trypanosomal DNA (potential mixed infections). Error bars represent the upper limit of the 95% CI

Overall, the *Trypanosoma* species diversity in tsetse gut samples was low. *Trypanosoma grayi* was the most prevalent species encountered at all sampling sites (Fig. 2a, Table 2, Additional file 2: Figure S1). Overall prevalence in the different sampling sites ranged from 6% (95% CI: 1.6–16.2%) in Ijah Gwari to 55.3% (95% CI: 48.2–62.2%) in Old Oyo NP (Table 2, Fig. 2a). An ITS1 amplicon length of approximate 320 bp is characteristic for *T. grayi*. However, amplicons in this size range could also be attributed to *T. simiae* (345 bp), *T. simiae* Tsavo (320 bp) or *T. theileri* (350 bp). Therefore, ITS1 and/or *gGAPDH* sequences of 35 samples that showed the 320 bp ITS1 fragment were analysed to confirm the *Trypanosoma* species present. All sequences shared high nucleotide identity with the respective *T. grayi* sequences (details on sequences and sequence origins can be found in Additional file 1: Table S1). Interestingly, a 500 bp amplicon was amplified together with *T. grayi* in half of the cases (51.6%, Additional file 3: Table S2). It is important to note that the 500 bp product was never detected alone.

*Trypanosoma congolense* was the second most prevalent *Trypanosoma* species detected in gut samples. Prevalence of *T. congolense* ranged from 3.2% (95% CI: 1.5–6.7%) in Old Oyo NP to 8.9% (95% CI: 3.5–20.7%) in Yankari GR (Fig. 2a, Table 2). ITS1 (6 samples) and/or *gGAPDH* (7 samples) sequence analysis of 12 representative samples from a total of 20 *T. congolense*-positive samples confirmed the species assignment and allowed the identification of all three subgroups described for *T. congolense*: the riverine/forest subgroup was predominant with 8 cases compared to the savannah subgroup (3 cases) and Kilifi subgroup in one case.

Besides *T. grayi* and *T. congolense*, *T. theileri* was identified by sequencing in two gut samples in Kainji Lake NP (assigned amplicon lengths 350 bp and 400 bp, respectively; Additional file 1: Table S1, Fig. 2a). It should be noted that during the initial screening the amplicon sizes from these two samples were distinct from the 320 bp PCR products from *T. grayi* samples. In four samples, ITS1 amplicons of 200 bp were detected and assigned to *T. vivax* (Additional file 1: Table S1).

Multiple bands were detected in 87 of the 174 *Trypanosoma*-positive samples. In 88.5% of these cases they were due to the presence of *T. grayi* with the unknown 500 bp amplicon (Additional file 3: Table S2), which might either represent a PCR-derived artefact or a different template within the samples. Apart from this combination, double amplicons representing *T. grayi* and *T. congolense* were obtained in 9 flies.

### Tsetse proboscis samples

*Trypanosoma vivax* was the most frequently encountered trypanosome in tsetse proboscis. Overall, 11.9% (95% CI: 9.2–15.5%) of all investigated flies carried *T. vivax* (Table 3). This was confirmed by sequencing part of the *gGAPDH* gene of a random subset of samples (14 of a total of 49). Strikingly, almost a third of all positive proboscis samples (28.6%) revealed an ITS1 amplicon size of 320 bp. Seven sequences obtained from 6 randomly chosen samples (2 by sequence analysis of ITS1, 5 by partial *gGAPDH* analysis; see Additional file 1: Table S1) were all related to *T. grayi*. Interestingly, in 50% of the flies that harboured *T. grayi* DNA in their proboscis no amplification was obtained in the gut (Additional file 3: Table S2). The presence of *T. grayi* in proboscis samples is unexpected, as the life-cycle of this parasite should be confined to the mid- and hindgut [25, 26]. Of note is that the unknown 500 bp amplicon was not observed in the proboscis.

**Table 3:** Prevalence (in %) of *Trypanosoma* species in the different collection sites in the tsetse proboscis

	All	Yankari GR	Kainji Lake NP	Old Oyo NP	Cross River NP	Ijah Gwari
*T. vivax*	11.7 (8.9–15.1)	14.6 (7.2–27.2)	12.8 (7.8–20.4)	6.6 (3.8–11.2)		28.3 (18–41.6)
*T. grayi*	5.4 (3.6–7.9)		1.8 (0.3–6.4)	7.2 (4.2–11.9)	19.0 (7.7–40.0)	5.7 (1.5–15.4)

*Notes*: *Trypanosoma* species were assigned according to ITS1 size [20, 21] and confirmed by sequencing of selected ITS1 and gGAPDH amplicons [22]. The lower and upper limits of the 95% confidence interval are indicated in parentheses

The only other amplicons detected in proboscis were low molecular weight amplicons of unknown origin (NI100) [20] in 3.6% of the samples. Additional attempts to subclone and sequence this amplicon failed. Additionally, no amplicons were obtained with primers with *gGAPDH*. Therefore, the samples were not analysed further and considered negative for trypanosomes.

Notably, *T. congolense* was not detected in any of the analysed proboscises.

### Differences in trypanosome distribution according to locations and fly species

The largest numbers of tsetse were collected in Old Oyo NP (*n* = 190), Kainji Lake NP (*n* = 111), Yankari GR (*n* = 49) and Ijah Gwari (combined sampling in March and November 2014: *n* = 53). In Cross River NP (*n* = 21), sampling of tsetse was restricted by environmental factors at the time of collections. The tsetse species composition at the sampling sites reflected the respective environmental conditions. In Yankari GR, *Glossina morsitans submorsitans* and *G. tachinoides* were caught in a similar ratio (53% and 47%, respectively). In Kainji Lake NP, *G. tachinoides* was more prevalent (86%) compared to *G. m. submorsitans*. In Old Oyo NP, Ijah Gwari and Cross River NP, *G. palpalis palpalis* was the predominant species caught. A detailed description on tsetse species distribution and unidentified *Glossina* species has already been given by Shaida et al. [18].

**Fig. 3:**
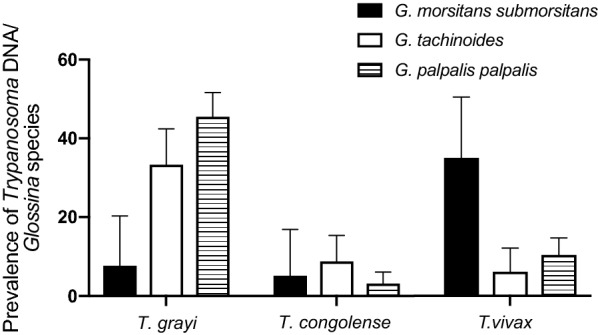
Prevalence of *T. grayi*, *T. congolense* and *T. vivax* in different *Glossina* species. Shown is the prevalence of the respective *Trypanosoma* species in all collected flies of one *Glossina* species, irrespective of the collection site. *Trypanosoma* species were assigned according to ITS1 size estimation [20, 21] and confirmed by sequencing of ITS1 and *gGAPDH* [22]. Error bars represent the upper limit of the 95% CI

*Trypanosoma grayi* was the most prevalent trypanosome detected in tsetse gut tissue in all sites visited, although its overall prevalence differed and was highest in Old Oyo NP, where more than half of the flies (55.3%) carried *T. grayi* DNA (Fig. 2a, Table 2). *Trypanosoma grayi* was predominantly detected in riverine species of tsetse (Fig. 3). In Yankari GR and Kainji Lake NP, the savannah species *G. m. submorsitans* and the riverine species *G. tachinoides* coexist. In these two locations, *T. grayi* was significantly more prevalent in *G. tachinoides* (*χ*^2^ = 9.739, *df* = 1, *P* = 0.0018).

The prevalence of *T. congolense* was highest in Yankari GR. It should be noted that at this site it was predominantly detected alone. In contrast, *T. congolense* was frequently detected together with *T. grayi* in Kainji Lake NP and Old Oyo NP. *Trypanosoma congolense* was not present in flies from Cross River NP, which might be due to the low sample size. Overall, no correlation between fly species and prevalence was found for *T. congolense* (Fig. 3).

*Trypanosoma vivax* was the predominant trypanosome detected in proboscis samples from Yankari GR, Kainji Lake NP and in Ijah Gwari (Fig. 2b). However, in Old Oyo NP *T. grayi* was found in proboscis as frequently as *T. vivax*. This is partially reflected by the prevalence in the different tsetse species; in contrast to *T. grayi*, *T. vivax* was detected predominantly in the savannah species *G. m. submorsitans* (35%) compared to *G. tachinoides* (6%) collected from the same locations (Fig. 3b).

### Phylogenetic relationships of Nigerian trypanosomes

**Fig. 4:**
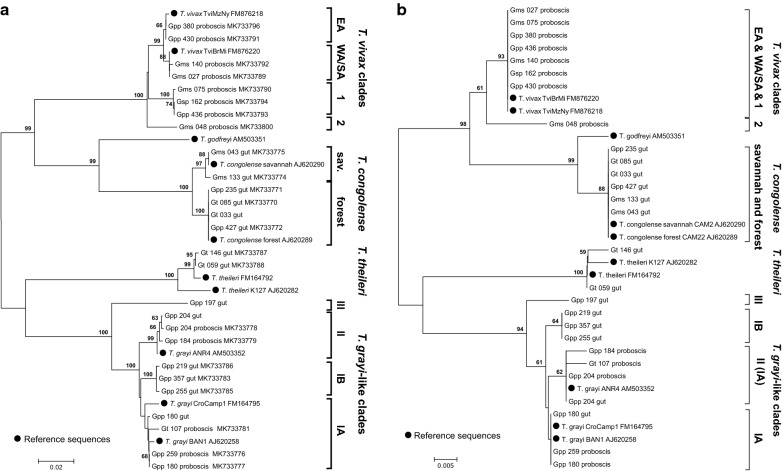
Phylogenetic analysis of *gGAPDH* sequences from trypanosome species detected in Nigerian tsetse. **a** *gGAPDH* nucleotide sequences of 27 field samples and 10 reference sequences were aligned as described under methods and a 677 bp stretch extracted. A Neighbour-Joining tree was calculated using complete gap deletion and tested with 700 bootstrap replications using MEGA6 [24]. **b** Phylogenetic relationships of the respective translated protein sequences were inferred by the Neighbour-Joining tree as described before. Note the diversity of *T. grayi* sequences on nucleotide and amino acid level. Reference sequences are marked by black circles and accession numbers are indicated after each sequence if available. Numbers of the field sequences reference the fly identification number. Gut and proboscis state the origin of the DNA amplicon. *Abbreviations*: EA, East Africa; WA, West Africa; SA, South America; Gpp, *G. palpalis palpalis*; Gms, *G. morsitans submorsitans*; Gt, *G. tachinoides*; Gsp, *Glossina* spp

Phylogenetic analysis of *gGAPDH* sequences from representative samples provided insight into the diversity of the trypanosomes circulating in the different study sites. The newly generated nucleotide sequences were aligned and phylogenetic relationships inferred together with reference sequences retrieved from GenBank (Fig. 4). *Trypanosoma congolense*, *T. vivax* and *T. godfreyi* clustered together, while *T. theileri* and *T. grayi* formed a second group, which is in accordance with previous analyses [22, 27]. The subgroups of *T. congolense* included in this analysis, *T. congolense* savannah and *T. congolense* forest, were found on the same branch, but formed two distinct clusters with strong bootstrap support. When the phylogenetic relationships were inferred from the respective translated protein sequences, both *T. congolense* subgroups formed a homogenous cluster and were not resolved (Fig. 4b). This was also observed for *T. vivax* clusters. While the respective *gGAPDH* sequences segregated into different clusters at the nucleotide sequence level (Fig. 4a, *T. vivax* clades), they formed a homogeneous group at the amino acid level (Fig. 4b). The exception is sample “Gms 048”, that grouped further away from the other *T. vivax* samples on both, DNA and protein level. It should be noted that two *T. vivax* sequences from Ijah Gwari (“Gpp 380” and “Gpp 430”) are closely related to East African isolates (EA). A third group composed of sequences obtained from various locations (Kainji Lake NP, Old Oyo NP, Ijah Gwari) did not group within previously described East African (clade EA) or West African/South American (clade WA/SA) isolates (Clade 1).

Interestingly, *T. grayi* samples showed an even higher diversity than that observed for *T. vivax* and *T. congolense*, the distances between the clusters were larger than for the very well described *T. congolense* savannah and forest subgroups. *Trypanosoma grayi* clustered in three groups (IA and IB, II and III); of these, group III was formed by only one sequence (“Gpp197”, Fig. 4a). Notably, when the sequences were analysed at the amino acid level, these groups are still similarly defined as at the nucleotide level (Fig. 4b).

## Discussion

This study provides an overview of trypanosome species in the gut and proboscis of tsetse from different regions in Nigeria, with a focus on protected areas (GR and NP). Using molecular tools, i.e. ITS1 amplification supported by *gGAPDH* analysis and sequencing, we detected a limited *Trypanosoma* species diversity throughout the sampling sites. Three trypanosome species were frequently detected, *T. grayi*, *T. vivax* and *T. congolense*. Additionally, *T. theileri* was identified in two tsetse flies.

In contrast to the limited species diversity, a high intraspecific genetic diversity was detected within *T. vivax* and *T. grayi* populations based on *gGAPDH* sequence analysis (Fig. 4).

Surprisingly, *T. grayi* was highly prevalent in all sampling sites and DNA indicating presence of this trypanosome was amplified from 37% of all tsetse gut samples (154 of 413, Table 2). This is striking as *T. grayi* was last reported in tsetse from Nigeria in 1924 [28]. A 500 bp ITS1-amplicon of unidentified origin was detected together with *T. grayi* in all locations except Ijah Gwari (Additional file 2: Figure S1).

Principally, the use of methods with high sensitivity is associated with the risk of detecting cross-contamination between samples, i.e. during dissections. To investigate this possibility, we analysed the sequence identity of several amplicons from subsequently dissected flies. However, the sequences analysed were different, indicating that the trypanosomal DNA did not come from one single sample, which would be expected if cross-contamination would have occurred. Inconsistent presence of either the 320 bp or 320/500 bp amplicons in subsequently dissected flies further indicates that cross-contamination is unlikely.

### Trypanosome prevalence in the different sampling sites

Four of the five sampling sites in our study were protected areas. We identified *T. congolense* and *T. vivax* in all locations except Cross River NP, emphasising the role of these protected areas as reservoirs for pathogenic trypanosomes. Livestock farming in close proximity to protected areas is therefore at risk of AAT transmission either due to dispersal of the flies during the wet season or during migration of livestock herds along the parks. Additionally, this finding also emphasises the problem faced in disease control programmes. Parasites circulating in wildlife within protected areas will interfere with elimination of the disease in country-wide approaches.

Previous surveys identified mainly *T. congolense* and *T. vivax* as animal pathogenic trypanosomes in Nigeria (summarised in [12]). Our data are in accordance with this finding. Interestingly, *T. vivax* was more prevalent in the northern parts of the country, with the highest prevalence in Ijah Gwari, Yankari GR and Kainji Lake NP, while in the southern Old Oyo NP prevalence was lowest (Table 3, Fig. 2b). Previously, a survey in cattle revealed the opposite, with a higher number of *T. vivax* infections in southern Nigeria [29].

In Yankari GR, a recent survey based on ITS1 analysis identified mainly *T. congolense* and *T. vivax*, co-circulating with a few cases of *T. godfreyi* and *T. simiae* [13]. However, they did not record *T. grayi*. Interestingly, the authors also found a higher infection rate in female tsetse [13], while we did not detect significant differences between sexes (Additional file 1: Table S1). Differences in prevalence and *Trypanosoma* species diversity might be explained by the use of different molecular tools or a different year and season of sampling.

No molecular data were previously available from areas within or adjacent to Kainji Lake NP. In a survey using microscopic identification in 2000, 13.6% of tsetse caught in Kainji Lake NP were found to be positive for trypanosomes, mainly identified as *T. vivax* or *T. congolense* [30]. However, it is difficult to compare these surveys, as the sensitivity and specificity of the methods used differ [31]. Kainji Lake NP was the only collection site where we detected *T. theileri*. This trypanosome is mostly transmitted by tabanids and *Stomoxys* spp. [1] and is found only occasionally in tsetse flies [32], explaining the overall low occurrence in the tsetse samples. Interestingly, a few cases of *T. vivax* were detected in gut tissue in Kainji Lake NP. Although the life-cycle of this species is restricted to the mouthparts, parasites can be detected in the foregut up to four days after an infectious blood meal [6], which might be the reason for this observation.

Similarly, molecular data on trypanosomes circulating in Cross River NP as well as Ijah Gwari have so far not been available. Despite the fact that sample numbers were low in Cross River NP, *T. grayi* and *T. vivax* were detected in several cases, although the latter was restricted to the gut indicating remains of a recent blood meal. The highest prevalence of *T. vivax* cases in the proboscis was recorded in Ijah Gwari where it was found co-circulating with *T. congolense*. This indicates that these two pathogenic parasites are prevalent in this area and pose a risk for livestock farmers.

In Old Oyo NP, prevalence of *T. grayi* was highest amongst all the sampling sites, while prevalence of *T. congolense* in the gut and *T. vivax* in the proboscis were lowest (Fig. 2, Tables 2, 3). In further studies it will be interesting to monitor the influence of *T. grayi* infections in tsetse on infections with another *Trypanosoma* sp. within the same vector.

Interestingly, *T. b. brucei* was highly prevalent in tsetse (approximately 50%) collected in close proximity to cattle farms in Oyo State, while *T. vivax* and *T. congolense* were detected less frequently [14]. During our surveys, we detected no DNA originating from *T. brucei* (*s.l.*), which is in agreement with previous reports that the occurrence of this parasite is low in Nigeria [13, 16, 33, 34]. These contradictory findings with respect to *T. b. brucei* prevalence, but also overall species diversity, might reflect seasonal or local differences of trypanosome populations, especially in close proximity to livestock farms.

Overall, the differences in trypanosome prevalence in the different locations might be due to the different *Glossina* species composition and animal reservoirs present. *Trypanosoma grayi* was detected in only a few cases in *G. m. submorsitans* (7%), while in *G. tachinoides* collected at the same locations it was detected in 34% (Fig. 4). These findings are in good agreement with previous reports that *G. morsitans* rarely feed on reptiles, among which are crocodiles, the predominant hosts of *T. grayi* [1, 7], while reptiles are a frequent food source for *G. tachinoides* and *G. palpalis* [35, 36]. Therefore, *T. grayi* might not encounter *G. morsitans*, which feed preferentially on suids and bovids. In accordance, Lloyd & Johnson [28] observed a correlation between presence of non-mammalian blood meal and *T. grayi* infection rates in tsetse between different sampling sites. During our collections, traps were set in close proximity to small streams, rivers or lakes that are potential habitats to crocodiles and other reptiles and crocodiles are widely distributed throughout all the NP and GR visited.

Vector competence might also be involved in the observed differences in prevalence between the sampling sites. For example, riverine species are considered less susceptible to *T. congolense* infections [37], which might explain the lack of mature *T. congolense* infections in proboscis samples from our study. Additionally, *T. congolense* forest, the predominant subgroup identified, has been shown to be less efficient in completing its life-cycle in tsetse irrespective of the *Glossina* species involved [37]. Accordingly, in a previous survey in Yankari GR, the authors noted that flies carrying *T. congolense* forest in the gut had not developed mature infections and that the savannah subgroup appeared to mature more efficiently to infective parasites present in the proboscis [13].

### Genetic diversity

One of the main findings in this study is the genetic diversity detected within trypanosome populations, especially for *T. grayi* and *T. vivax*. *Trypanosoma vivax* clustered in four clades at the nucleotide sequence level (Fig. 4a). One clade included West African and South American *T. vivax*-types (WA/SA) as observed previously [38, 39]. Sequences from Ijah Gwari shared high similarity with East African isolates, possibly indicating the dynamic movement of these strains across the continent. However, two more clades were identified that share less similarity with any described West or East African *T. vivax* strains. Clade 1 is widely spread throughout Nigeria and was found in Kainji Lake NP, Old Oyo NP and Ijah Gwari. Clade 2 is represented by only one sequence in this study. It was obtained from a *G. m. submorsitans* in Yankari GR (Gms048) and shared lower (96–97%) sequence identity with all other *T. vivax* sequences. This sequence clustered away from all other *T. vivax* representatives at both nucleotide and amino acid sequence level (Fig. 4a, b). It should be further investigated whether this might be a representative of *T. uniforme*, a member of the *Duttonella* that had been frequently identified in bovines and other ungulates in Uganda and Democratic Republic of Congo in the early 20th century [40], but has been out of focus in recent decades [39]. Otherwise, together with the other clades it might resemble different *T. vivax* subgroups that are circulating in Nigeria. Generally, West African isolates are considered more pathogenic than East African isolates [1]. In fact, the pathogenicity of *T. vivax* has been shown to be strain- (or subgroup-) dependent as observed for isolates of the same geographical region [41]. Additionally, the genetic diversity between clades 1 and 2 on the one hand and the West and East African clades on the other hand is comparable to the diversity found within *T. congolense* subgroups (forest, savannah and Kilifi) that also show different virulence [42] and different vector preferences [37, 43]. Thus, it seems justified to speculate that the diversity of the *T. vivax* clades might also translate into different pathogenicity or host range.

In comparison to *T. vivax* and *T. congolense*, *T. grayi* appears to be genetically even more diverse and the *T. grayi*-like sequences clustered into 3 clades that were defined at the nucleotide and amino acid level (Fig. 4a, b). A similar diversity as found here for *T. grayi* has been described for *T. theileri*. Generally considered non-pathogenic, *T. theileri* was found to cluster in distinct clades with recent indications of clade-dependent pathogenicity in cattle (Paguem et al., personal communication, [20, 44, 45]). Additionally, it is surprising that DNA from *T. grayi* was detected in the proboscis of 22 tsetse, an observation contrasting the described life-cycle [25]. In our field collections, it was not possible to investigate the proboscis during dissection for live parasites. Therefore, we had to rely on the molecular data obtained. Recently, ITS1 amplicons derived from *T. grayi* were also amplified from the proboscis of two *G. m. submorsitans* in Cameroon [20]. These findings alone might be explained by residual DNA from a recent blood meal or even a contamination during collection or dissection. However, two studies in Northern Cameroon also identified *T. grayi* DNA in cattle (Paguem et al., personal communication, [20]). This further emphasises that *T. grayi* has to be monitored closely.

The genetic diversity of *T. vivax* and *T. grayi* indicates genetically dynamic populations. The question remains, what is driving this genetic diversity and at what point is the genetic diversity an indicator for changes in pathogenicity or host tropism? In the case of *T. brucei*, transfer of a single gene can determine whether it is pathogenic to humans, and this transfer has been proven to occur during mating between human pathogenic and animal pathogenic subspecies [46]. It is not known which factors determine the host range and pathogenicity of animal pathogenic trypanosomes and mating has been proven only within two trypanosome species based on mating experiments or microsatellite data [46–48]. The genetic diversity observed here might be an indication for such events, and further research should investigate this possibility.

## Conclusions

The prevalence and species diversity of trypanosomes was investigated in Nigerian tsetse flies. Although *T. brucei gambiense* was not identified, corresponding with previous reports of the low occurrence of HAT in Nigeria [11], animal pathogenic trypanosomes were widely distributed in the flies. The main concern is *T. vivax* as a diverse and widely distributed pathogen. *Trypanosoma congolense* was frequently detected in tsetse gut tissue, but not in the mouthparts. Nevertheless, it is prevalent and thus animal reservoirs must be present. Yankari Game Reserve and Ijah Gwari harboured the highest prevalence of these two species and thus provide reservoirs for AAT. An unexpected finding was the high prevalence of *T. grayi* in all areas surveyed, with high levels reaching up to 55% in selected areas. The fact that this is, to our knowledge, the first report on the presence of *T. grayi* in Nigerian tsetse flies in recent years might indicate that this parasite has previously been overlooked. The high genetic diversity observed for *T. vivax* and *T. grayi* is alerting. These parasite populations have to be monitored closely to follow their geographical distribution and to elucidate their pathogenicity in potential new hosts to actively prevent outbreaks of AAT and to detect any potential changes in life-cycle and pathogenicity.

## Supplementary information

**Additional file 1: Table S1.** Overview of all PCR analysis including all sequences generated from the tsetse samples (gut and proboscis). Displayed are the date and site of collection, the unique fly ID (fly #), sex and morphological species. Flies were dissected in the field in order of appearance. If applicable, molecular *Glossina* species identification is indicated separately. Sheet one (gut) includes all data on molecular analysis of trypanosomes in tsetse gut samples, including all obtained sequences and all screenings with specific primers. Sheet two (proboscis) includes all data on molecular analysis of trypanosomes in tsetse proboscis samples.

**Additional file 2: Figure S1.** Absolute number of *Trypanosoma* cases in tsetse gut and proboscis within the respective sampling locations. The ITS1 region of Kinetoplastida was amplified, and all samples showing one or more amplicons were considered positive for trypanosomes. Species were assigned according to their ITS1-amplicon size as described before. The absolute numbers of cases are displayed.

**Additional file 3: Table S2.** Trypanosome species present in respective tsetse gut and proboscis samples according to ITS1 amplicon size [20, 21]. Number of flies with one (single), two (double) or three (triple) ITS1 amplicons detected in the gut (horizontal) are correlated with the amplicons detected in the respective proboscis of the same fly (vertical). *Trypanosoma* species were assigned according to the respective length of the ITS1 amplicon. *Abbreviations*: *Tv*, *T. vivax*; *Tg*, *T. grayi*; *Tth*, *T. theileri*; *Tc*, *T. congolense*; 500 bp, unknown 500 bp amplicon.

## Data Availability

All data generated or analysed during this study are included in this published article and its additional files. Representative *gGAPDH* sequences have been deposited in the NCBI database with accession numbers MK733770–MK733800. Full length ITS1 sequences have been deposited in the NCBI database with accession numbers MK756201–MK756203.

## Abbreviations

AAT: African animal trypanosomiasis
gGAPDH: glycosomal glyceraldehyde-3-phosphate dehydrogenase
GR: Game Reserve
HAT: human African trypanosomiasis
ITS1: internal transcribed spacer 1
NP: National Park

## References

[1] Hoare CA (1972). The trypanosomes of mammals.

[2] Desquesnes M, Dia ML (2003). *Trypanosoma vivax*: mechanical transmission in cattle by one of the most common African tabanids, *Atylotus agrestis*. Exp Parasitol..

[3] Desquesnes M. Livestock trypanosomoses and their vectors in Latin America. Paris, France: OIE (World Organization for Animal Health); 2004.

[4] Cayla M, Rojas F, Silvester E, Venter F, Matthews KR (2019). African trypanosomes. Parasit Vectors..

[5] Dyer NA, Rose C, Ejeh NO, Acosta-Serrano A (2013). Flying tryps: survival and maturation of trypanosomes in tsetse flies. Trends Parasitol..

[6] Ooi C-P, Schuster S, Cren-Travaillé C, Bertiaux E, Cosson A, Goyard S (2016). The cyclical development of *Trypanosoma vivax* in the tsetse fly involves an asymmetric division. Front Cell Infect Microbiol..

[7] Hoare CA (1929). Studies on *Trypanosoma grayi* II. Experimental transmission to the crocodile. Trans R Soc Trop Med Hyg..

[8] Minter-Goedbloed E, Pudney M, Kilgour V, Evans DA (1982). First record of a reptile trypanosome isolated from *Glossina pallidipes* in Kenya. Z. Parasitenkd..

[9] Fisher AC, Schuster G, Cobb WJ, James AM, Cooper SM, León AA (2013). Molecular characterization of *Trypanosoma* (*Megatrypanum*) spp. infecting cattle (*Bos taurus*), white-tailed deer (*Odocoileus virginianus*), and elk (*Cervus elaphus canadensis*) in the United States. Vet Parasitol..

[10] National Population Estimates. National Population Commission and National Bureau of Statistics Estimates, Abuja. 2006. http://nigerianstat.gov.ng/elibrary, Population and Migration, Population 2006-2016. Accessed 14 Mar 2019.

[11] World Health Organization. Number of new reported cases (*T. b. gambiense*). 2018. http://apps.who.int/gho/data/node.main.A1636?lang=en. Accessed 2 Mar 2019.

[12] Odeniran PO, Ademola IO (2018). A meta-analysis of the prevalence of African animal trypanosomiasis in Nigeria from 1960 to 2017. Parasit Vectors..

[13] Isaac C, Ciosi M, Hamilton A, Scullion KM, Dede P, Igbinosa IB (2016). Molecular identification of different trypanosome species and subspecies in tsetse flies of northern Nigeria. Parasit Vectors..

[14] Odeniran PO, MacLeod ET, Ademola IO, Welburn SC (2019). Molecular identification of bloodmeal sources and trypanosomes in *Glossina* spp., *Tabanus* spp. and *Stomoxys* spp. trapped on cattle farm settlements in southwest Nigeria. Med Vet Entomol..

[15] Onyekwelu KC, Ejezie FE, Eze AA, Ikekpeazu JE, Ezeh RC, Edeh GC (2017). Prevalence of trypanosome infection in tsetse flies from Oji River and Emene axis of Enugu State, Nigeria: a preliminary report. Trop Parasitol..

[16] Majekodunmi AO, Fajinmi A, Dongkum C, Picozzi K, Thrusfield MV, Welburn SC (2013). A longitudinal survey of African animal trypanosomiasis in domestic cattle on the Jos Plateau, Nigeria: prevalence, distribution and risk factors. Parasit Vectors..

[17] Odeniran PO, Macleod ET, Ademola IO, Welburn SC (2019). Molecular identification of bovine trypanosomes in relation to cattle sources in southwest Nigeria. Parasitol Int..

[18] Shaida SS, Weber JS, Gbem TT, Ngomtcho SCH, Musa UB, Achukwi MD (2018). Diversity and phylogenetic relationships of *Glossina* populations in Nigeria and the Cameroonian border region. BMC Microbiol..

[19] Challier A, Laveissière C (1973). Un nouveau piège pour la capture des glossines (*Glossina*: Diptera, Muscidae): description et essais sur le terrain. Cah ORSTOM Série Entomol Méd Parasitol..

[20] Ngomtcho SCH, Weber JS, Ngo Bum E, Gbem TT, Kelm S, Achukwi DM. Molecular screening of tsetse flies and cattle reveal different *Trypanosoma* species including *T. grayi* and *T. theileri* in northern Cameroon. Parasit Vectors. 2017;10:631. 10.1186/s13071-017-2540-7 PMC5747950 29287598

[21] Adams ER, Malele II, Msangi AR, Gibson WC (2006). Trypanosome identification in wild tsetse populations in Tanzania using generic primers to amplify the ribosomal RNA ITS-1 region. Acta Trop..

[22] Hamilton PB, Stevens JR, Gaunt MW, Gidley J, Gibson WC (2004). Trypanosomes are monophyletic: evidence from genes for glyceraldehyde phosphate dehydrogenase and small subunit ribosomal RNA. Int J Parasitol..

[23] Drummond AJ, Ashton B, Buxton S, Cheung M, Cooper A, Heled J, et al. Geneious v5.5. 2011. http://geneious.com.

[24] Tamura K, Stecher G, Peterson D, Filipski A, Kumar S (2013). MEGA6: molecular evolutionary genetics analysis version 6.0. Mol Biol Evol..

[25] Hoare CA (1931). Studies on *Trypanosoma grayi* III. Life-cycle in the tsetse-fly and in the crocodile. Trans R Soc Trop Med Hyg..

[26] Hoare CA (1931). The peritrophic membrane of *Glossina* and its bearing upon the life-cycle of *Trypanosoma grayi*. Trans R Soc Trop Med Hyg..

[27] Hamilton P, Adams E, Njiokou F, Gibson W, Cuny G, Herder S (2009). Phylogenetic analysis reveals the presence of the *Trypanosoma cruzi* clade in African terrestrial mammals. Infect Genet Evol..

[28] Lloyd L, Johnson WB (1924). The trypanosome infections of tsetse-flies in northern Nigeria and a new method of estimation. Bull Entomol Res..

[29] Takeet MI, Fagbemi BO, Donato MD, Yakubu A, Rodulfo HE, Peters SO (2013). Molecular survey of pathogenic trypanosomes in naturally infected Nigerian cattle. Res Vet Sci..

[30] Ahmed AB, Omoogun GA, Shaida SS. Trypanosome infections in *Glossina* species at the Kainji Lake National Wild Life Park, Nigeria. In: Dike MC, Ajayi O, Okunade SO, Okoronkwo NO, Abba AA, editors. Entomology in nation building the Nigerian experience. Proceedings of ESN 30th Annual Conference held at Kano, Nigeria, 4th–7th October 1999. Zaria, Nigeria: CABI; 2000. p. 57–62.

[31] Adams ER, Hamilton PB (2008). New molecular tools for the identification of trypanosome species. Future Microbiol..

[32] Votýpka J, Rádrová J, Skalický T, Jirků M, Jirsová D, Mihalca AD (2015). A tsetse and tabanid fly survey of African great apes habitats reveals the presence of a novel trypanosome lineage but the absence of *Trypanosoma brucei*. Int J Parasitol..

[33] Jordan AM (1964). Trypanosome infection rates in *Glossina morsitans submorsitans* Newst. in northern Nigeria. Bull Entomol Res..

[34] Ahmed AB (2007). High trypanosome infections in *Glossina palpalis palpalis* Robineau-Desvoidy 1830 in Southern Kaduna State, Nigeria. Sci World J..

[35] Weitz B (1963). The feeding habits of *Glossina*. Bull World Health Organ..

[36] Clausen PH, Adeyemi I, Bauer B, Breloeer M, Salchow F, Staak C (1998). Host preferences of tsetse (Diptera: Glossinidae) based on bloodmeal identifications. Med Vet Entomol..

[37] Reifenberg JM, Cuisance D, Frezil JL, Cuny G, Duvallet G (1997). Comparison of the susceptibility of different *Glossina* species to simple and mixed infections with *Trypanosoma* (*Nannomonas*) *congolense* savannah and riverine forest types. Med Vet Entomol..

[38] Takeet MI, Fagbemi BO, Peters SO, DeDonato M, Yakubu A-M, Wheto M (2016). Genetic diversity among *Trypanosoma vivax* strains detected in naturally infected cattle in Nigeria based on ITS1 of rDNA and diagnostic antigen gene sequences. J Parasit Dis..

[39] Adams ER, Hamilton PB, Rodrigues AC, Malele II, Delespaux V, Teixeira MMG (2010). New *Trypanosoma* (*Duttonella*) *vivax* genotypes from tsetse flies in East Africa. Parasitology..

[40] Hoare CA, Broom JC (1938). Morphological and taxonomic studies on mammalian trypanosomes: IV Biometrical study of the relationship between *Trypanosoma uniforme* and *T. vivax*. Trans R Soc Trop Med Hyg..

[41] Osório ALAR, Madruga CR, Desquesnes M, Soares CO, Ribeiro LRR, da Costa SCG (2008). *Trypanosoma* (*Duttonella*) *vivax*: its biology, epidemiology, pathogenesis, and introduction in the New World - a review. Mem Inst Oswaldo Cruz..

[42] Bengaly Z, Sidibe I, Boly H, Sawadogo L, Desquesnes M (2002). Comparative pathogenicity of three genetically distinct *Trypanosoma congolense*-types in inbred BALB/c mice. Vet Parasitol..

[43] Ravel S, Grébaut P, Mariani C, Jamonneau V, Cuisance D, Gooding RH (2004). Monitoring the susceptibility of *Glossina palpalis gambiensis* and *G morsitans morsitans* to experimental infection with savannah-type *Trypanosoma congolense*, using the polymerase chain reaction. Ann Trop Med Parasitol..

[44] Garcia HA, Rodrigues AC, Martinkovic F, Minervino AHH, Campaner M, Nunes VLB (2011). Multilocus phylogeographical analysis of *Trypanosoma* (*Megatrypanum*) genotypes from sympatric cattle and water buffalo populations supports evolutionary host constraint and close phylogenetic relationships with genotypes found in other ruminants. Int J Parasitol..

[45] Jaimes-Dueñez J, Triana-Chávez O, Mejía-Jaramillo AM (2018). Spatial-temporal and phylogeographic characterization of *Trypanosoma* spp. in cattle (*Bos taurus*) and buffaloes (*Bubalus bubalis*) reveals transmission dynamics of these parasites in Colombia. Vet Parasitol..

[46] Gibson W, Peacock L, Ferris V, Fischer K, Livingstone J, Thomas J, Bailey M (2015). Genetic recombination between human and animal parasites creates novel strains of human pathogen. PLoS Negl Trop Dis..

[47] Morrison LJ, Tweedie A, Black A, Pinchbeck GL, Christley RM, Schoenefeld A (2009). Discovery of mating in the major African livestock pathogen *Trypanosoma congolense*. PLoS One..

[48] Peacock L, Ferris V, Bailey M, Gibson W (2008). Fly transmission and mating of *Trypanosoma brucei brucei* strain 427. Mol Biochem Parasitol..

