# Supplementary figures and images for: Genetic diversity of trypanosome species in tsetse flies (Glossina spp.) in Nigeria
Source: Parasit Vectors. 2019 Oct 14;12:481. doi: 10.1186/s13071-019-3718-y (PMC6792248; doi:10.1186/s13071-019-3718-y)

**Yankari GR**

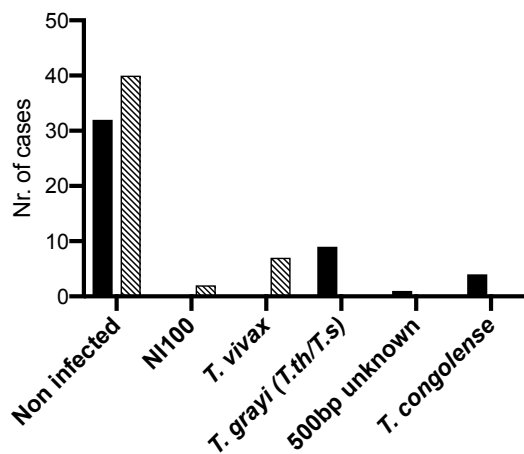

**Kainji Lake NP**

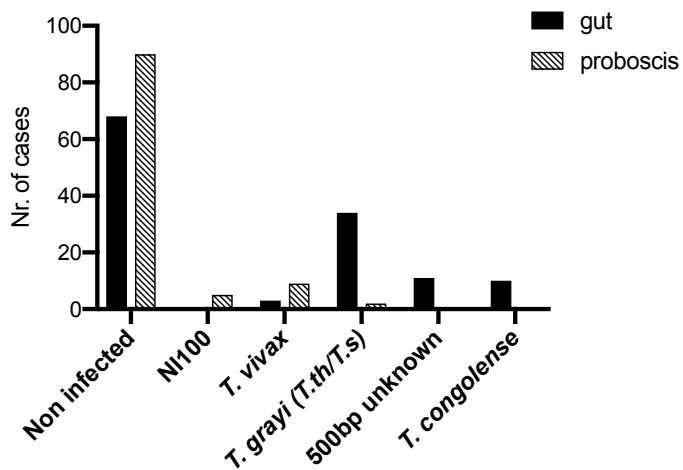

**Old Oyo NP**

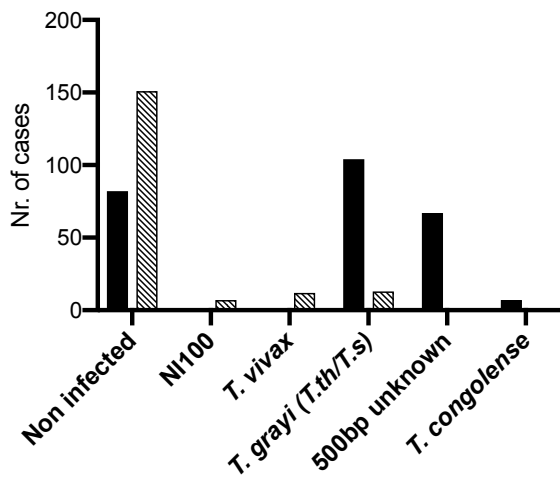

**Cross River NP**

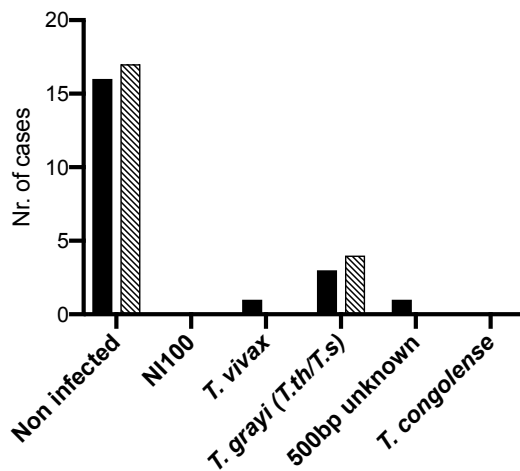

**Ijah Gwari I (03/14)**

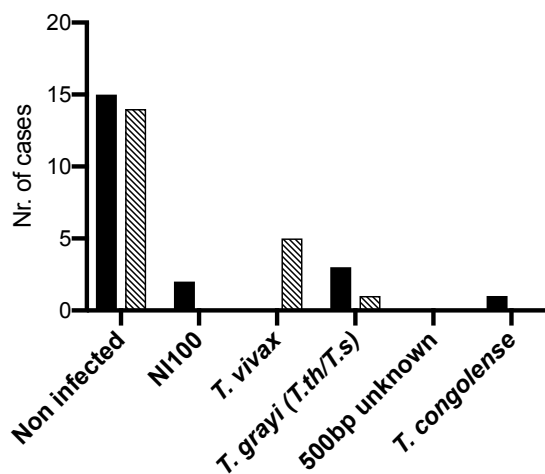

**Ijah Gwari II (12/14)**

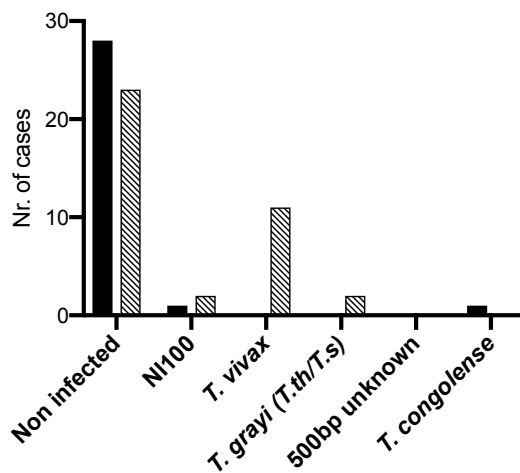

Supplement: Supplementary file 2 — Additional file 2: Figure S1. Absolute number of Trypanosoma cases in tsetse gut and proboscis within the respective sampling locations. The ITS1 region of Kinetoplastida was amplified, and all samples showing one or more amplicons were considered positive for trypanosomes. Species were assigned according to their ITS1-amplicon size as described before. The absolute numbers of cases are displayed. [file 13071_2019_3718_MOESM2_ESM.pdf]
