# Supplementary material for: Genetic diversity of trypanosome species in tsetse flies (Glossina spp.) in Nigeria
Source: Parasit Vectors. 2019 Oct 14;12:481. doi: 10.1186/s13071-019-3718-y (PMC6792248; doi:10.1186/s13071-019-3718-y)
Supplement: Supplementary file 3 — Additional file 3: Table S2. Trypanosome species present in respective tsetse gut and proboscis samples according to ITS1 amplicon size [20, 21]. Number of flies with one (single), two (double) or three (triple) ITS1 amplicons detected in the gut (horizontal) are correlated with the amplicons detected in the respective proboscis of the same fly (vertical). Trypanosoma species were assigned according to the respective length of the ITS1 amplicon. Abbreviations: Tv, T. vivax; Tg, T. grayi; Tth, T. theileri; Tc, T. congolense; 500 bp, unknown 500 bp amplicon. [file 13071_2019_3718_MOESM3_ESM.docx]

|  | |  |  | **ITS1 amplicon in proboscis** | | | | | | | | |  |
| --- | --- | --- | --- | --- | --- | --- | --- | --- | --- | --- | --- | --- | --- |
|  | |  |  | **Single amplicons** | | | **Double amplicons** | | |  | |  |  |
|  | |  |  | **NI100^a^** | ***Tv*** | ***Tg*** | **NI100^a^ /*Tv*** | ***Tv/Tv*^b^** | ***Tv/Tg*** | **No ITS1 amplicon (proboscis)** | **N. a.^c^** | | **Total** |
| **ITS1 amplicon in gut** | **Single amplicons** | | **NI100^a^** |  | 1 |  |  |  |  | 3 |  | | 4 |
|  |  |  | ***Tv*** |  |  |  |  |  |  | 3 |  | | 3 |
|  |  |  | ***Tg*** | 1 | 5 | 4 | 1 |  |  | 57 | 1 | | 69 |
|  |  |  | ***Tth*** |  | 1 |  |  |  |  |  |  | | 1 |
|  |  |  | ***Tc*** |  | 2 |  | 1 |  |  | 7 |  | | 10 |
|  | **Double amplicons** | | ***Tg*/ 500bp** | 2 | 3 | 7 | 1 |  |  | 58 | 5 | | 76 |
|  |  |  | ***Tg*/ *Tc*** |  |  |  |  |  |  | 5 |  | | 5 |
|  |  |  | ***Tth*/ *Tc*** |  |  |  |  |  |  | 1 |  | | 1 |
|  | **Triple amplicons** | | ***Tv*/ *Tc* /*Tc*^b^** |  |  |  |  |  |  | 1 |  | | 1 |
|  |  |  | ***Tg* /500bp/ *Tc*** |  |  |  |  |  |  | 3 |  | | 3 |
|  |  |  | ***Tg*/ *Tc*/ *Tc*^b^** |  |  |  |  |  |  | 1 |  | | 1 |
|  |  | | **No ITS1 amplicon (gut)** | 6 | 24 | 9 | 3 | 1 | 2 | 189 | 6 | | 239 |
|  |  | | **N.a.^c^** |  | 3 |  |  |  |  | 8 |  | | 11 |
|  |  | | **Total** | 9 | 39 | 20 | 6 | 1 | 2 | 335 | 12 | | 424 |

^a^NI100 consists of amplicons of a size between 100 and 150 bp and has not yet been identified [20]

^b^Double bands detected within one fly corresponding to the same species are indicated by *Tv/Tv* or *Tc/Tc*, respectively

*** From 23 of the analysed tsetse only gut or proboscis, respectively, was available for analysis. A total of 12 proboscis samples and 11 gut samples were lost during dissection or sample preparation and are marked as not available “N. a.”
